# The Effect of Mesenchymal Stem Cells on Dry Eye in Sjogren Syndrome Mouse Model

**DOI:** 10.3390/ijms24021039

**Published:** 2023-01-05

**Authors:** Soojung Shin, Seul-Gi Yoon, Miso Kim, Eun Jeong Cheon, Youngseo Jeon, Hyun Jung Lee, So-Hyang Chung

**Affiliations:** 1 Department of Ophthalmology and Visual Science, Catholic Institute of Visual Science, Seoul St. Mary’s Hospital, College of Medicine, The Catholic University of Korea, Seoul 06591, Republic of Korea; 2 Department of Biochemical Engineering, Seoil University, Seoul 06591, Republic of Korea

**Keywords:** autophagy, dry eye, lacrimal gland, Catholic MASTER Cells (hMSCs), Sjögren’s syndrome (SS), SS mouse model

## Abstract

Sjögren’s syndrome (SS) is a systemic autoimmune disease delineated by chronic lymphocytic infiltrates into the lacrimal or salivary glands, leading to severe dry eye and dry mouth. Mesenchymal stem cells have been shown to be effective in treating numerous autoimmune diseases. This study aimed to illustrate the effects of mesenchymal stem cells on the attenuation of dry eyes (DE) through the inhibition of autophagy markers in a SS mouse model. NOD/ShiLtJ female mice with developed DE were treated with either subconjunctival or lacrimal gland injections of hMSCs (Catholic MASTER Cells). After maintenance for 14 days, clinical DE markers such as tear secretion and corneal staining were observed, as well as goblet cell counts in the conjunctiva, infiltration of inflammatory foci, B and T cells, and autophagy markers in the lacrimal glands. Proinflammatory cytokine expressions of the cornea and conjunctiva, as well as the lacrimal glands, were examined. Clinical markers, such as tear secretion and corneal stain scores, goblet cell counts in the conjunctiva, and foci infiltrations in the lacrimal glands were attenuated in mice treated with subconjunctival or lacrimal gland injections of hMSCs compared to the PBS-treated control group. B cell marker B220 decreased in the lacrimal glands of hMSCs-treated mice, as well as reduced proinflammatory cytokine expressions in the lacrimal glands and cornea. Notably, expression of autophagy markers ATG5 and LC3B-II, as well as HIF-1α and mTOR which play roles in the pathways of autophagy modulation, were shown to be attenuated in the lacrimal glands of hMSCs-treated mice compared to the PBS-treated control mice. Treatment with hMSCs by lacrimal gland or subconjunctival injection demonstrated the alleviation of DE through the repression of autophagy markers, suggesting the therapeutic potentials of hMSCs in a SS mouse model.

## 1. Introduction

Sjögren’s syndrome (SS) is a progressive autoimmune disease that is distinguished by a deficiency in secretions of exocrine glands and the infiltration of lymphocytes, leading to severe dry eye (DE) and dry mouth [1]. The complexity of the pathogenesis of SS has resulted in many challenges in the process of endeavoring to discover treatments for the disease. Significant strides have been made in illuminating the roles of B cells and other immune modulations in the progression of SS, but despite the development of very severe DE in SS, treatments consist of nonspecific anti-inflammatory eyedrops which do little to confer effects on the chronic pathogenesis of the disease itself [2,3]. Without sufficient etiological treatment for this disease, there is a limit to the extent to which patients can be treated, and management of the symptoms remains palliative at best [4,5]. Thus, it is imperative from a clinical perspective to discover novel approaches for the treatment of SS DE.

In line with this, the most widely utilized form of adult stem cells is mesenchymal stem cells (MSCs) because they are able to differentiate into cells of varying lineages and confer therapeutic effects. MSCs are multipotent adult stem cells that are usually isolated from the bone marrow, umbilical cord, dental pulp, and other various tissues [6]. In part, the recent interest that MSCs have garnered in the medical community is largely because they were shown to not only modulate cell proliferation and tissue regeneration, but also exhibit immunosuppressive and anti-inflammatory properties [7,8]. Numerous MSCs studies have been conducted for their attenuating effects on various autoimmune-related diseases [9,10,11,12]. Of note, the research conducted on the application of MSCs in inflammatory ocular surface diseases illustrated its therapeutic potential and how it might be harnessed for the future [13,14,15]. One of the mechanisms of MSC-mediated immunomodulation was demonstrated in myocardial infarction to have a correlation with autophagy [16].

Of interest, previous studies conducted by our group revealed that autophagy markers were elevated in primary SS DE in comparison with the non-SS DE group, and the potential of tear ATG5 as a potential diagnostic biomarker for SS DE was indicated [17,18]. Moreover, chloroquine, which is used in clinical settings for the treatment of SS, was found to inhibit DE progression in a SS mouse model with a reduction of autophagy markers in the lacrimal glands [19].

In light of these findings, we aimed to demonstrate the effects of the administration of MSCs by either lacrimal gland or subconjunctival injection on the alleviation of DE by the inhibition of autophagy in a SS mouse model in this study.

## 2. Results

### 2.1. Clinical DE Parameters and Goblet Cell Counts Are Improved in hMSCs-Treated Mice

**Figure 1:**
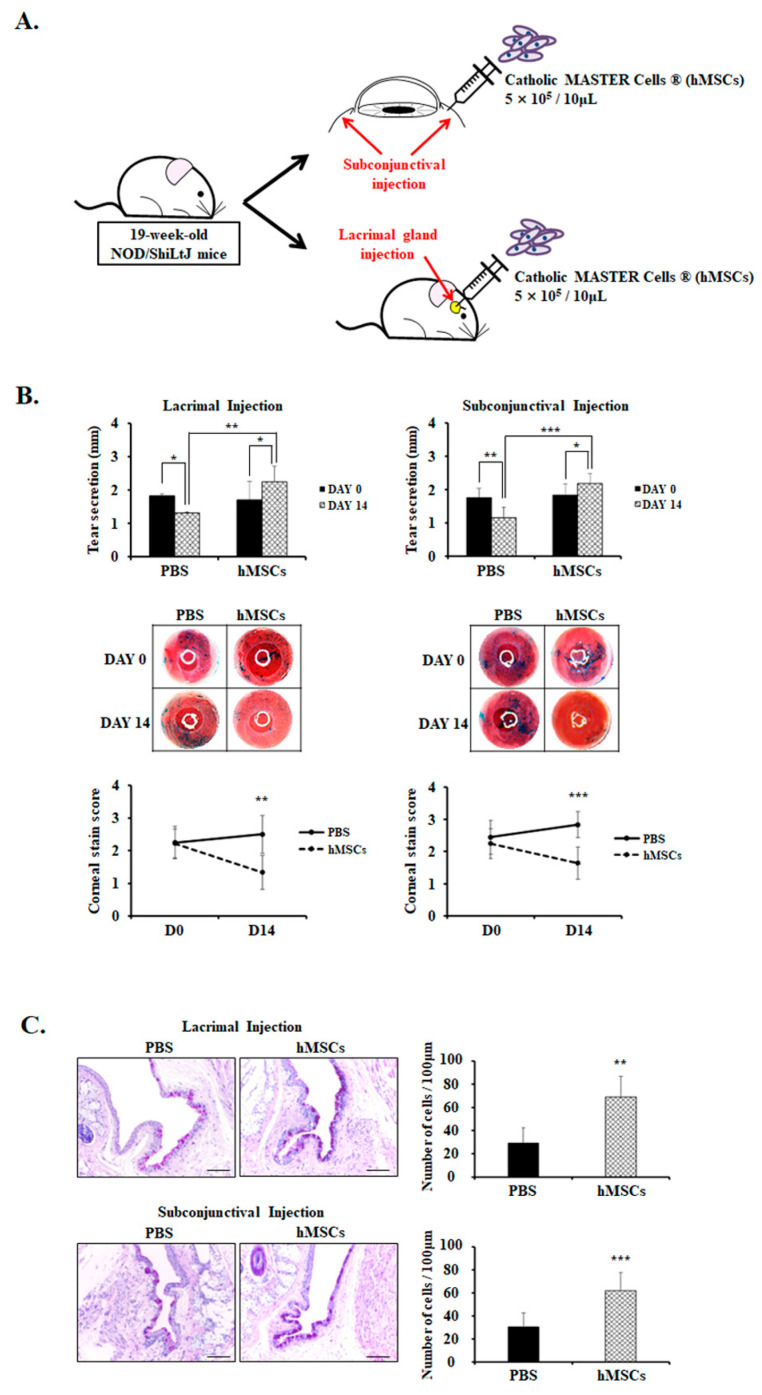
Analysis of clinical parameters and goblet cell count for PBS-treated or hMSCs-treated SS mouse model. (**A**) Schematic representation of the administration of the hMSCs through either the subconjunctival or lacrimal gland injection in 19-week-old NOD/ShiLtJ mice. (**B**) Tear secretion volume for mice treated with PBS or hMSCs by lacrimal gland or subconjunctival injection. Tear volume was assessed by the phenol red thread test on day 0 and day 14. The corneal stain scores were evaluated by lissamine green staining, as shown by representative photomicrographs. (**C**) Representative PAS staining photographs of the conjunctiva signifying the goblet cell density and numbers. Scale bar: 50 μm. Data are presented from six independent experiments (*n* = 6). * *p* < 0.05, ** *p* < 0.01, *** *p* < 0.001.

hMSCs were injected once by either lacrimal gland or subconjunctival injection and sacrificed after 14 days (Figure 1A). Clinical DE parameters such as tear production and corneal stain scores were measured on day 0 and day 14 (Figure 1B). The data showed that tear secretion was significantly higher in the hMSCs-treated group by lacrimal gland injection compared to the PBS-treated control group on day 14 (hMSCs = 2.24 ± 0.47; PBS = 1.31 ± 0.03, *p* < 0.01). Tear secretion significantly increased on day 14 compared to day 0 in the hMSCs-treated group by lacrimal gland injection (day 0 = 1.71 ± 0.56; day 14 = 2.24 ± 0.47, *p* < 0.05), whereas tear secretion decreased in the PBS-treated control group (Day 0 = 1.82 ± 0.07; day 14 = 1.31 ± 0.03, *p* < 0.05) because of the natural progression of disease in the SS mouse model. Similarly, mice treated with subconjunctival injection of hMSCs demonstrated a significant increase in tear secretion compared to the PBS-treated control group on day 14 (hMSCs = 2.18 ± 0.29; PBS = 1.15 ± 0.32, *p* < 0.001). Tear secretion significantly increased on day 14 compared to day 0 (day 0 = 1.83 ± 0.34; day 14 = 2.18 ± 0.29, *p* < 0.05), and demonstrated the reduction of tear secretion in the PBS-treated control group (day 0 = 1.75 ± 0.29; day 14 = 1.15 ± 0.32, *p* < 0.01). The corneal stain scores by lissamine green staining were rated on a scale from 0 to 3 by blind observers, with higher numbers indicating increased severity. As shown in Figure 1B, corneal stain scores on Day 14 were significantly lower in the hMSCs-treated group by lacrimal gland injection compared to the PBS-treated mice (hMSCs = 1.33 ± 0.52; PBS = 2.50 ± 0.58, *p* < 0.01). Likewise, mice treated with hMSCs by subconjunctival injection exhibited notable attenuations in corneal stain scores, with a score of 1.64 ± 0.50 compared with 2.83 ± 0.41 (*p* < 0.001) in PBS-treated control mice.

Additionally, conjunctival goblet cells significantly decreased in the PBS-treated control mice with the progression of SS disease phenotype with aging, but demonstrated significant goblet cell count recovery with subconjunctival or lacrimal gland injection of hMSCs (*p* < 0.01) (Figure 1C).

### 2.2. Lacrimal Gland Inflammatory Foci Decreased in hMSCs-Treated Mice

**Figure 2:**
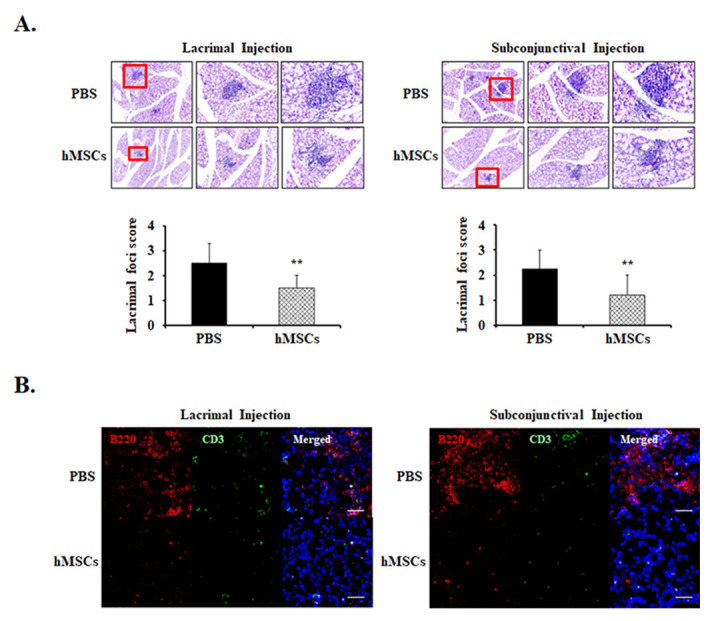
Representative lacrimal gland histology for PBS-treated or hMSCs-treated mice. (**A**) Lacrimal gland histological sections obtained from 19-week-old NOD/ShiLtJ mice stained with H&E. Extraorbital lacrimal glands were removed and fixed in formalin then serially sectioned and stained. The representative photomicrographs demonstrate the leukocyte infiltrations of the lacrimal glands and their pertaining lacrimal gland foci score. (**B**) The effects of hMSCs on B-cell and T-cell markers were shown with representative photomicrographs stained with B220 and CD3, respectively. Scale bar: 1000 μm. Data are presented from six independent experiments (*n* = 6). ** *p* < 0.01.

To investigate the effects of subconjunctival or lacrimal gland injection of hMSCs in the lacrimal glands of SS mice, histologic section examinations were conducted. Lacrimal gland histology by H&E staining demonstrated multiple foci of leukocytic infiltrates in the PBS-treated group in the development of SS DE. Meanwhile, it was observed that mice treated with lacrimal gland or subconjunctival injection of hMSCs demonstrated significant amelioration of foci infiltration in the lacrimal glands, with a score of 1.50 ± 0.52 (*p* < 0.01) and 1.21 ± 0.78 (*p* < 0.01), respectively (Figure 2A).

Immunofluorescent staining for B cell marker anti-B220 and T cell marker anti-CD3 were observed in the lacrimal glands. As can be seen from the results, it was revealed that expression levels of B220^+^ were notably increased in the PBS-treated control mice, whereas treatment of hMSCs conferred attenuation in the infiltration of these cells (Figure 2B). However, the expression levels of CD3 were shown to be comparatively low without significant differences in both the PBS and hMSCs-treated mice.

### 2.3. hMSCs-Treated Mice Demonstrated Autophagy Marker Attenuations in the Lacrimal Glands

**Figure 3:**
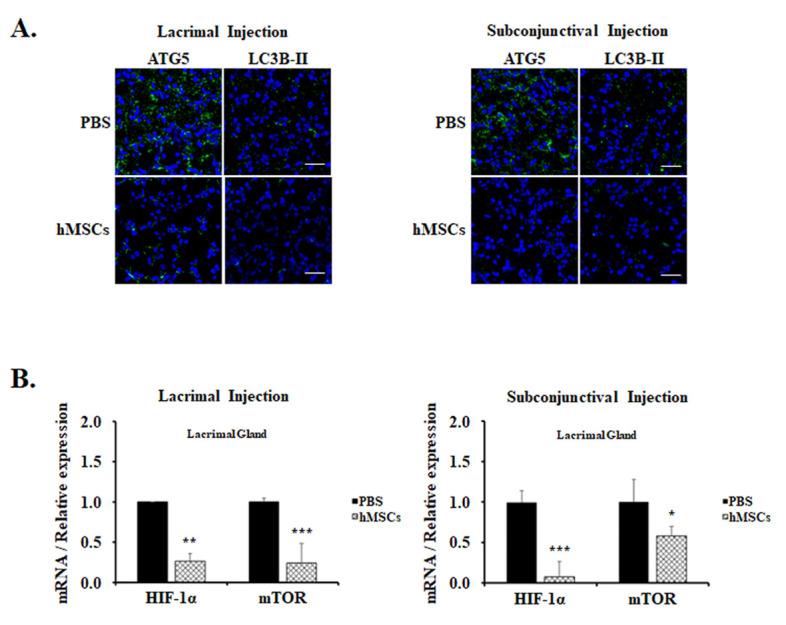
Autophagy expression in the LG of PBS-treated or hMSCs-treated SS mouse model. (**A**) hMSCs effects on autophagy induction shown for lacrimal glands stained with autophagy markers ATG5 or LC3B-II. Scale bar: 20 μm. (**B**) Gene expression levels of markers related to autophagy as shown in the lacrimal glands of hMSCs or PBS-treated mice by lacrimal gland or subconjunctival injection. Data are presented from six independent experiments (*n* = 6). * *p* < 0.05, ** *p* < 0.01, *** *p* < 0.001.

For the examination of the effect of hMSCs treatment on autophagy in mice, immunofluorescent staining with autophagy markers anti-ATG5 and anti-LC3B-II Abs was examined in the lacrimal glands. These autophagy markers are expressed as punctate cytoplasmic staining patterns, and it was observed to be prominent in the lacrimal glands of PBS-treated control mice (Figure 3A). Contrarily, autophagy markers in the lacrimal glands of mice treated with hMSCs were significantly mitigated, suggesting the suppression of autophagy induction. Moreover, HIIF-1α and mTOR, which play important roles in the pathway of autophagy modulation, were also shown to be significantly decreased in the gene expression levels in the lacrimal glands of hMSCs-treated mice compared to the PBS-treated control group (Figure 3B).

### 2.4. Expression of Proinflammatory Cytokines Decreased in hMSCs-Treated Mice

**Figure 4:**
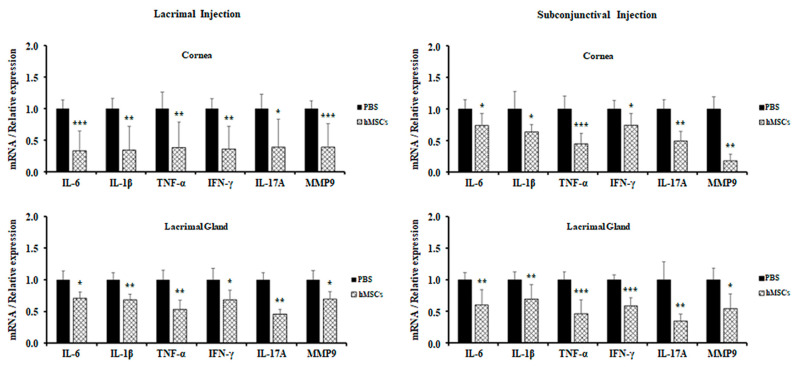
Gene expression of inflammatory cytokines in the cornea and LG of PBS-treated and hMSCs-treated mice. Gene expression levels of inflammatory cytokines were examined in the cornea and conjunctiva and in the lacrimal glands of mice treated with hMSCs or PBS. Data are presented from six independent experiments (*n* = 6). * *p* < 0.05, ** *p* < 0.01, *** *p* < 0.001.

For further evaluation of the role of hMSCs in a SS mouse model, expression levels of proinflammatory cytokines in the cornea and lacrimal glands were observed by real-time PCR. It was derived from the results that hMSCs-treated mice showed attenuations in the gene expression levels of proinflammatory cytokines for both the cornea and the lacrimal glands. All proinflammatory cytokine gene expressions (IL-6, IL-1β, TNF-α, IFN-γ, IL-17A, and MMP9) were significantly decreased in the cornea and lacrimal gland of mice treated with lacrimal gland or subconjunctival injection of hMSCs (Figure 4).

## 3. Discussion

The current study imparted the effects of hMSCs on the alleviation of DE through the inhibition of autophagy in a SS mouse model. The hallmarks of the SS DE, such as infiltration of lymphocytes to the lacrimal glands, were demonstrated to be attenuated in mice treated with hMSCs by lacrimal gland or subconjunctival injection. Furthermore, clinical phenotypes were markedly shown to be reduced by increased tear secretions and decreased corneal epithelial defects, as well as recovery in the number of goblet cells and attenuations of proinflammatory cytokines. Numerous studies have been conducted to explicate pathological mechanisms of SS through animal models to better understand the progression of the disease [5,13,20,21]. As SS is a progressive autoimmune disease, this entails that the detection of early disease markers and the design of therapeutics based on those findings is a key aspect to further understanding its systemic manifestations. Our findings indicate that the corneal epithelial defects were attenuated in mice treated with hMSCs, as well as demonstrating increased tear secretion and goblet cell recovery in the conjunctiva. This suggests that hMSCs have the potential to preserve and ultimately recover the clinical DE signs experienced with SS disease progression.

MSCs, with their capacity for self-renewal and immunomodulation abilities, have been utilized as potential therapeutic treatments for various maladies, such as autoimmune diseases [14,22,23,24]. MSCs were shown to inhibit the differentiation of antigen-presenting cells, as well as the proliferation of B cells and T cells [25]. Numerous studies have been conducted on the effects of MSCs treatment in mouse models of SS, with MSCs derived from the bone marrow of mice and administered via the intravenous or intraperitoneal injection pathways. Xu et al. (2012) demonstrated that treatment of allogenic MSCs in female NOD mice alleviated experimental SS disease development by immunoregulatory pathways such as suppressing Th17 and Tfh responses and restoration of salivary gland secretory function [14]. Similarly, treatment of MSCs in female NOD mice demonstrated a reduction of lymphocytic infiltrations in the salivary glands, as well as a prevention of the loss of saliva flow [26]. Moreover, intraperitoneal injections of mouse bone marrow-derived MSCs (BD-MSCs) in NOD mice revealed a decrease in the size of the lymphocytic foci in the treated mice compared to the control group, as well as an increase in aquaporin 5 levels, resulting in increased tear production in NOD mice with the treatment of BD-MSCs [13]. Abughanam et al. (2019) administered bone marrow-derived MSCs or MSC-extracts into NOD mice, with the results showing perseverance of lacrimal and salivary gland functions compared to the control group, higher levels of Il-10 and Treg, and inhibition of B220^+^ B cells [27]. These studies are in line with our results that showed attenuations in the infiltration of B220^+^ B cells in the lacrimal glands, as well as recovery of tear production and corneal epithelial surfaces with the lacrimal or subconjunctival injection of human bone marrow-derived MSCs in a SS mouse model. Of interest, both subconjunctival and lacrimal gland injections of hMSCs exhibited reductions of B cell infiltrations in the lacrimal glands.

Moreover, evidence from studies on SS mouse models has shown that the degradation and dysfunction of exocrine glands with the infiltration of inflammatory cells, such as B cells, are one of the dominant traits of SS pathogenesis [28,29,30,31]. Of note, it could be observed that B cells were predominantly infiltrated in the lacrimal glands of SS mice compared to the T cells, denoting the crucial underlying role of B cells in the development of SS disease pathogenesis. Our results showed a B cell dominant role in the pathological mechanism of hMSCs for the recovery of DE parameters in the SS mouse model, with attenuated infiltration of inflammatory cells in the lacrimal glands, most prominently the B cell marker, B220, but not the T cell marker, CD3. Previous studies have also shown the correlation between B cells and SS disease, demonstrating lower levels of the B-cell activating factor (BAFF) in the salivary glands of SS mice treated with MSCs, indicating that MSCs confer their therapeutic effects through the attenuations of B cells in the lymphocytic infiltrates [26,27]. Taken together, this strongly supports the evidence that human-derived MSCs showed potential in conferring therapeutic roles for SS DE.

In terms of disease pathogenesis for SS, much is still needed to clearly pinpoint its mechanisms. There have been few studies conducted on the role of autophagy in SS disease progression [32,33]. Recently, our group has previously demonstrated the potential of autophagy marker ATG5 as a therapeutic and diagnostic biomarker, observing the autophagy marker’s increase in primary SS DE compared to non-SS DE [17,18]. Additionally, our group has further shown that autophagy was induced in the early stages of the SS mouse model, and treatment of autophagy inhibitor, chloroquine, conferred the inhibition of SS disease progression [19]. While studies conducted on the role of B cells on MSCs and SS DE disease pathways are in abundance, literature on the effects MSCs confers in alleviating the SS DE disease pathway through autophagy has been comparatively rarely investigated. As can be observed from this study, it was interesting to note that autophagy markers ATG5 and LC3B-II were shown to be alleviated in lacrimal glands of mice treated with MSCs by both lacrimal and subconjunctival injections. Other studies have shown that stemness and differentiation capacities of the MSCs were regulated by autophagy, including organ function repairing effects of MSCs [16]. Jakovljevic et al. (2018) noted that autophagy induction might play a significant role in the differentiation capacity of MSCs, indicating that modulation of autophagy could enhance its immunosuppressive characteristics [34].

There have been copious amounts of studies conducted demonstrating the anti-inflammatory effects of MSCs when applied to ocular surface inflammatory disorders that include, but are not limited to, dry eye disease, allergic eye diseases, and chemical eye burn [35,36,37]. It was shown that the application of bone marrow-derived MSCs in NOD mice as an SS animal model attenuated lymphocyte infiltration in the salivary glands, as well as preventing loss in salivary flow rate. Not only that, the MSC-treated group demonstrated decreased levels of proinflammatory cytokines such as IFN-γ and TGF-β, suggesting therapeutic effects of MSC in SS diseases [38]. Application of bone marrow-derived MSC into the intraorbital gland showed reductions in the IFN-γ and Il-2 cytokines in an inflammation-induced dry eye model of mice [15]. In another study, MSC administration by subconjunctival injection in a rat model of corneal alkali burns showed reductions in the corneal infiltration of inflammatory cells [39]. Likewise, our studies have demonstrated that hMSCs may confer their anti-inflammatory effects by suppressing the pro-inflammatory cytokines in the lacrimal glands and the cornea.

There were also potential limitations in this current study. This experiment was conducted utilizing bilateral injections of hMSCs in either the lacrimal gland or the subconjunctiva of the SS mouse model, meaning that the contralateral eye was not used for internal controls. Rather, a separate experimental group was considered by injecting PBS bilaterally as a control to the SS mouse model. Therefore, an interesting future research direction to consider would be to conduct injections of hMSCs in a SS mouse model by mode of unilateral injections and observe the differences in the contralateral eye used as an internal control, this would offer causality based on local experimental interventions.

In conclusion, this study edified the therapeutic effects of hMSCs on SS DE through the attenuation of autophagy markers. The current study utilized bone marrow-derived Korean FDA-approved hMSCs by administration via lacrimal gland or subconjunctival injection. As was observed from the results, both the subconjunctival and the lacrimal gland injection method of administering hMSCs were promising in attenuating SS DE in a SS mouse model. The subconjunctival injection method demonstrated similar marked declines of foci infiltrations and inflammatory cells in the lacrimal glands compared to the lacrimal injection method. This has many implications in terms of clinical applications, there it is easier to administer the hMSCs by a non-invasive subconjunctival injection in patients rather than by the invasive lacrimal gland injection method. Thus, it can be surmised that future advancements in the application of hMSCs to patients will most likely be in the form of the subconjunctival injection method. In all, the results from this research offer an illuminating glimpse into the potential of the hMSCs in alleviating SS DE and may offer a starting point to guide future clinical possibilities on the usage of hMSCs for the treatment of SS in patients.

## 4. Methods and Materials

### 4.1. Mouse Model of SS

This study utilized 19-week-old non-obese diabetic NOD/ShiLtJ female mice as a mouse model for SS, for it was indicated from previous studies to be the stage when SS develops and progression of DE occurs [19]. They were purchased from Jackson Laboratories (Bar Harbor, ME, USA) and maintained under pathogen-free conditions in an environment with a 12-h/12-h light-dark cycle. The mice received sterilized food and water ad libitum at the animal facilities in the Catholic University of Korea (Seoul, Republic of Korea). The procedures conducted in this study all adhered to the ARVO Statement for the Use of Animals in Ophthalmic and Vision Research protocol and were approved by the Institutional Animal Care and Use Committee.

### 4.2. hMSCs Treatment

Mice were treated with hMSCs (Catholic MASTER Cells) derived from human bone marrow supplied from the Catholic Institute of Cell Therapy and approved by the Korean FDA as well as toxicology/safety tests to be clinically applicable. They were stored in DMEM low (with 20% FBA), and the certificate of analysis stated cell viability levels of over 80%. hMSCs were cultured, then collected, centrifuged, and the number of cells counted. Then, the appropriate number of hMSCs cells needed per injection or eyedrop volume in mice were calculated and washed, then added to a set PBS volume. hMSCs were administered once by lacrimal gland or subconjunctival injection with a concentration of 5 × 10^5^/10 μL and the same volume of PBS was injected for the PBS-treated control group, as demonstrated in Figure 1A. The administration of either hMSCs or PBS was conducted as a blind study. The mice were then maintained for 14 days and sacrificed.

### 4.3. Phenol Red Thread Test

The assessment of tear production was conducted with phenol red impregnated cotton threads (Zone-Quick; Menicon, Nagoya, Japan) that were administered bilaterally into the lateral canthus of the conjunctival fornix of mice for 60 s. After, a millimeter scale ruler was used to measure the length of the cotton thread that became wet. The data from both eyes from each animal was averaged.

### 4.4. Corneal Surface Staining

The severity of the progression of DE in the mouse model of SS was evaluated by observing and scoring the stained corneal surface. A single drop of 3% Lissamine Green B (Sigma-Aldrich Corp., St. Louis, MO, USA) was administered into the inferior lateral conjunctival sac of mice. Then, the evaluation of the clinical parameters of the corneal surface integrity with lissamine green staining was executed in a blinded manner with a standardized scoring system ranging from 0 to 3. A score of 0 indicated no punctate staining, score 1 indicated less than one-third of the cornea stained, score 2 indicated two-thirds or less of the cornea stained, and score 3 indicated more than two-thirds of the cornea stained [32].

### 4.5. Periodic Acid Schiff (PAS) Staining for Conjunctival Goblet Cell Histology

For the assessment of conjunctival goblet cell histology, whole eyeballs of mice that included the superior and inferior forniceal conjunctiva were harvested and fixed in formalin. Thereafter, the samples were set in paraffin blocks and sliced in transversal planes through the superior and inferior conjunctival fornices into 4-μm thick sections and then stained with PAS (Abcam, Cambridge, UK). After staining, four different cuts per 100 μm of the stained eyes were counted from the same mice and the average goblet cell count for each eye was calculated in terms of goblet cell density.

### 4.6. Histologic Analysis of Lacrimal Glands

The lacrimal glands of mice were extracted and fixed in formalin. Then, they were embedded and cut into horizontal sections that were 4-μm thick. The cut sections underwent dewaxing by xylene for 40 min and then subsequent hydrations by serial immersions of 100%, 95%, 90%, 80%, and 70% ethanol, then PBS. After, the lacrimal gland sections were stained with hematoxylin and eosin and then observed under a microscope at X200 and X400. The grading for the foci was executed using the protocol described previously [27,40,41]. Lacrimal gland immunofluorescence was observed by microwaving the slides in target retrieval solution (Target Retrieval Solution; DAKO, Carpinteria, CA, USA) for 15 min and washing three times for 2 min each with PBS with 0.05% Tween (PBST). Lacrimal gland slides were then incubated with blocking buffer (10% normal goat serum in PBST) for 1 h and incubated with LC3B-II (NovusBio), anti-ATG5 (NovusBio, Littleton, CO, USA), CD3 (Santa Cruz Biotechnology, Dallas, TX, USA), B220 (BD Biosciences, San Jose, CA, USA), or LC3B-II (Santa Cruz Biotechnology, Dallas, TX, USA) Abs in PBST overnight at 4˚C. After the slides were washed with PBST, they were incubated with AlexaFluor 546 or 488-conjugated anti-mice or rabbit IgG Ab, then washed with PBST again and mounted with DAPI (Vectashield; Vector Laboratories, Burlingame, CA, USA) mounting medium. Following that, the slides were viewed with confocal microscopy (Zeiss LSM 800 with Airyscan; Carl Zeiss Meditec, Oberkochen, Germany).

### 4.7. RNA Isolation and Real-Time PCR

Total RNA was extracted from the LG and corneas of mice with TRIzol reagent (Gibco-Invitrogen, Grand Island, NY, USA). After, reverse transcriptase (SuperScript III; Promega, Madison, WI, USA) was used for complementary DNA (cDNA) synthesis, and the real-time PCR method was initiated using SYBR Green I (Takara Bio Inc., Kusatsu, Shiga, Japan), with housekeeping gene GAPDH as the internal calibration for the average threshold cycle value of desired target genes. Relative quantitation was observed by the 2^−ΔΔCt^ method. Primer sequences utilized in this study are indicated in Table 1.

### 4.8. Statistical Analysis

The statistical significance between the groups was observed through a nonparametric, two-tailed Mann–Whitney *U* test using statistical software (GraphPad Prism software 9.1.1, La Jolla, CA, USA). The *p* < 0.05 value was regarded as significant, *p* < 0.01 as highly significant, and *p* < 0.001 as extremely highly significant. The data shown in this study are representative of three independent experiments that were conducted with six mice in each group.

## Figures and Tables

**Table 1:** Sequence list of primers used in real-time PCR.

Gene	Forward Primer Sequence (5’-3’)	Reverse Primer Sequence (5’-3’)
GAPDH	GGT CCT CAG TGT AGC CCA AG	AAT GTG TCC GTC GTG GAT CT
IL-6	AGT TGC CTT CTT GGG ACT GA	TTG GGA GTG GTA TCC TCT GTG
IL-1β	CAG GCA GGC AGT ATC ACT CA	AGG TGC TCA TGT CCT CAT CC
TNF-α	CAC CTG GCC TCT CTA CCT TG	TGG TCA CCA AAT CAG CGT TA
IFN-γ	GCT TTA ACA GCA GGC CAG AC	GGA AGC ACC AGG TGT CAA GT
IL-17A	TTC AGG GTC GAG AAG ATG CT	AAA CGT GGG GGT TTC TTA GG
MMP9	AGG TGG ACC ATG AGG TGA AC	CGG TTG AAG CAA AGA AGG AG
HIF-1α	GTC GGA CAG CCT CAC CAA ACA G	TAG GTA GTG AGC CAC CAG TGT CC
mTOR	CTC AAG CGA TCC AGT TGT CA	AGA AGG TGG GGA CAC TGA TG

## Data Availability

Data is contained within the article.
